# Using social cognition models to understand why people, such as perfectionists, struggle to respond with self‐compassion

**DOI:** 10.1111/bjso.12531

**Published:** 2022-03-09

**Authors:** Marios Biskas, Fuschia M. Sirois, Thomas L. Webb

**Affiliations:** ^1^ 7315 Department of Psychology The University of Sheffield Sheffield UK

**Keywords:** perfectionism, prototype willingness model, self‐compassion, self‐regulation, theory of planned behaviour

## Abstract

Responding with self‐compassion to lapses in goal pursuit helps people to achieve their goals, yet evidence suggests that some people struggle to respond with self‐compassion. The current research proposes that social cognition models such the Theory of Planned Behaviour and the Prototype Willingness Model could explain why some people, such as those high in perfectionistic concerns, struggle to respond with self‐compassion. We therefore conducted a pre‐registered prospective study that measured participants’ beliefs about self‐compassion, difficulties enacting self‐compassionate responding, perfectionistic concerns, and then tested their ability to be self‐compassionate in response to a recalled and future lapse. The results showed that participants were less likely to respond with self‐compassion to lapses if they held negative beliefs about self‐compassion and experienced difficulties enacting self‐compassion. Participants high in perfectionistic concerns were more likely to have negative beliefs about self‐compassion and experience difficulties enacting self‐compassion. Together, these findings provide evidence that social cognition models can be used to understand self‐compassionate responding and identify why some people struggle to respond with self‐compassion to goal lapses.

## INTRODUCTION

Most important goals (e.g., exercising regularly, meeting work deadlines) require continued efforts over a lengthy period of time, with the consequence that people inevitably encounter lapses and setbacks. Research has demonstrated that how people respond to these lapses influences whether they achieve their goals. Specifically, people who respond with self‐kindness, connectedness, and mindfulness (i.e., self‐compassion; Neff, 2003) are more likely to successfully pursue their goals (e.g., Miyagawa et al., 2018), while those who respond with self‐criticism are more likely to abandon their goals (e.g., Powers et al., 2007). We suggest that how people respond to lapses can be viewed like other responses and behaviours (e.g., whether people make healthy food choices or become angry with a colleague) and therefore that social cognition models, such as the Theory of Planned Behaviour (TPB; Ajzen, 1991) and the Prototype Willingness Model (PWM; Gibbons et al., 1998), can help to explain whether people will respond to lapses with self‐compassion.

Self‐compassion has been conceptualized in a number of different ways. According to Neff (2003), self‐compassion involves taking a kind, non‐judgmental, and accepting stance towards oneself in times of perceived failure or difficulty. This perspective views self‐compassion as consisting of three bipolar dimensions: (i) treating oneself with kindness versus being self‐judgmental, (ii) seeing personal mistakes as common to humanity versus feeling isolated, and (iii) being mindful of one's feelings versus over‐identifying with them. Other models of self‐compassion, such as that described by Gilbert (2010), focus on compassion as a quality that can ‘flow’ from self to others, others to self, or to self from self in the form of self‐compassion. From this perspective, self‐compassion is characterized as first noticing one's suffering and then responding to it with a motivation to take action to reduce suffering, whilst tolerating difficult emotions in an accepting and non‐judgemental manner.

Growing evidence has demonstrated that self‐compassion promotes well‐being and facilitates self‐regulation and goal striving. For example, interventions designed to promote self‐compassion increase happiness and life satisfaction, as well as reduce stress and depression (Ferrari et al., 2019; Kirby et al., 2017). Research also indicates that self‐compassion helps people to regulate their levels of physical activity, eating, and sleep (Adams & Leary, 2007; Magnus et al., 2010; Sirois et al., 2015), motivates persistence following failure (Breines & Chen, 2012), and promotes adjustment after failing to achieve a goal (Miyagawa et al., 2018).

### The challenges of being self‐compassionate

Despite the benefits of self‐compassion, there is emerging evidence that some people have negative views about being self‐compassionate or may even fear being self‐compassionate. For example, prior work suggests that some people believe that cultivating a self‐compassionate stance will lower their personal standards, decrease their motivation to grow as a person, and thus fail to achieve their goals (Kelly et al., 2021). Additionally, some people may associate being self‐compassionate with negative traits such as weakness, selfishness, irresponsibility, or self‐indulgence (Bayir & Lomas, 2016; Campion & Glover, 2017; Kelly et al., 2021; Robinson et al., 2016; Simpson et al., 2021). Furthermore, some people may believe that being self‐compassionate goes against the expectations of their family or culture (Bayir & Lomas, 2016; Campion & Glover, 2017; Gilbert, 2019), or that they do not deserve to experience self‐compassion (Gilbert et al., 2011). Finally, there is preliminary evidence that some people lack confidence in their ability to be self‐compassionate, are unfamiliar with the concept of self‐compassion (Campion & Glover, 2017; Kelly et al., 2021; Pauley & McPherson, 2010), or tend (and sometimes even prefer) to respond with self‐criticism when they experience difficulties (Bayir & Lomas, 2016; Kelly et al., 2021; Tobin & Dunkley, 2021).

Negative views towards being self‐compassionate may explain why some people struggle to respond with self‐compassion (Chwyl et al., 2020). People with high levels of perfectionistic concerns (hereafter, PC) are a case in point. PC is a higher order form of perfectionism that appears to capture the most maladaptive aspects of perfectionism, as it entails harsh critical and negative self‐evaluations, hypersensitivity to others’ evaluations, excessive concern over mistakes, and an inability to experience satisfaction even when successful (Sirois & Molnar, 2016). People high in PC relentlessly strive to achieve their goals to meet the high standards imposed by themselves or others (Flett & Hewitt, 2006; Spence & Robbins, 1992). People high in PC take a ‘zero tolerance’ stance towards their performance, with single lapses interpreted as signs of complete failure. They then blame themselves for (what they perceive to be) their substandard performance (Curran & Hill, 2018; Stoeber et al., 2008), with the consequence that they may subsequently abandon their goal (Sirois et al., 2017). Not surprisingly, evidence has demonstrated that people high in PC tend to have lower levels of (trait) self‐compassion (e.g., Stoeber et al., 2020; Tobin & Dunkley, 2021) and are less likely to respond to difficulties with self‐compassion (Lizmore et al., 2017). There is also evidence from clinical research that people high in self‐criticism, a key component of PC, struggle with self‐compassion because they fear being self‐compassionate (Gilbert & Procter, 2006).

### Using social cognition models to understand self‐compassionate responding

The aforementioned work points to potential reasons why some people struggle to respond with self‐compassion. A precise social cognitive framework for organizing and integrating this evidence would therefore provide the basis for testing specific hypotheses regarding the processes involved. The work of Gilbert and colleagues (Gilbert et al., 2011; Gilbert & Procter, 2006) takes a primarily clinical view of self‐compassion and focuses mainly on people's affective responses to self‐compassion, by proposing, for example, that people's fears (i.e., negative affective responses) are a key barrier to being self‐compassionate. From this perspective, difficulties with self‐compassion are addressed via therapeutic interventions such as compassion‐focused therapy (Gilbert, 2010) designed to aid clinically ‘at risk’ populations.

A social cognitive approach to understanding the barriers to being self‐compassionate can provide an overview of the challenges to being self‐compassionate in day‐to‐day life, identify conceptual similarities between these challenges, as well as provide testable hypotheses that can be used to systematically investigate the factors that influence self‐compassionate responding. The current research aims to offer such an integrative theoretical framework. Specifically, we propose that how people respond to lapses can be viewed like any other behaviour and thus that social cognition models such as the TPB and PWM might help to understand self‐compassionate responding and why some people, such as those high in PC, struggle to respond to lapses in goal pursuit with self‐compassion.

### Theory of planned behaviour

The TPB posits that behaviour is a function of the direction and strength of people's intentions (e.g., I strongly intend to respond with self‐compassion) which, in turn, is a function of their behavioural, normative, and control beliefs (Ajzen, 1991; Fishbein & Ajzen, 2010). Behavioural beliefs reflect the person's evaluations of the behaviour in terms of how it makes them feel (termed affective attitudes; e.g., I believe that responding with self‐compassion would be unpleasant/pleasant) and what they think about the behaviour (termed cognitive attitudes; e.g., I believe that responding with self‐compassion would be harmful/beneficial). Normative beliefs refer to the degree to which people believe that significant others in their life would approve of their engaging in the behaviour (termed injunctive norms; e.g., my parents think that I should respond with self‐compassion) and actually engage in the behaviour (termed descriptive norms; e.g., my parents respond with self‐compassion). Finally, control beliefs refer to the extent to which people believe that they have control over performing the behaviour (e.g., I am confident that I can respond with self‐compassion) and predict behaviour directly, as well as predicting what people intend to do. Considerable evidence has indicated that behavioural, normative, and control beliefs predict a variety of behaviours (Armitage & Conner, 2001; Hagger et al., 2002).

The TPB views behavioural, normative, and control beliefs as the sole determinants of intentions and behaviour, with all other factors (e.g., personality traits, demographic variables) viewed as distal predictors that influence behaviour through their influence on these three constructs (i.e., this is known as ‘the sufficiency assumption;’ Ajzen, 1991). For example, people with higher levels of PC may be less likely to respond with self‐compassion because they are more likely to have negative beliefs about self‐compassionate responding. However, evidence has shown that some personality traits may directly predict behaviour above and beyond the factors specified in the TPB. For example, extraversion can predict exercise behaviour over and above the factors specified in the TPB (Hoyt et al., 2009; Rhodes et al., 2005). It is therefore important to measure relevant individual differences (e.g., levels of PC) alongside beliefs in order to evaluate whether and how they are related to behaviour (e.g., directly and/or indirectly, via the beliefs specified in social cognition models like the TPB).

There is also evidence that additional beliefs may predict intentions and behaviour over and above the beliefs proposed by the TPB. For example, research has demonstrated that the degree to which people believe that they have a moral obligation to engage in a particular behaviour (termed personal norms; e.g., I personally feel that I should respond with self‐compassion) can predict intentions to perform the behaviour above and beyond the beliefs specified in the TPB (Heath & Gifford, 2002; White et al., 2009). Furthermore, there is some evidence suggesting that the extent to which people believe that others in their culture engage in the behaviour (termed cultural norms; e.g., People from my culture respond with self‐compassion) may increase the predictive power of the TPB (Cha et al., 2007; Iakovleva, 2016). Given this evidence and prior work suggesting that some people think that being self‐compassionate goes against the expectations of their society and culture (e.g., Campion & Glover, 2017), we extended the TPB by examining personal and cultural norms with respect to self‐compassionate responding.

A final consideration with respect to using the TPB to predict self‐compassionate responding, is that the TPB posits a direct relationship between intentions and behaviour, assuming that people have control over the behaviour. However, research has shown that intentions may not always lead to a change in behaviour (i.e., this is known as ‘the intention‐behaviour gap;’ Sheeran, 2002; Sheeran & Webb, 2016; Webb & Sheeran, 2006). That is, although people may have positive beliefs about a behaviour and intend to engage in that behaviour, they may still struggle to perform the behaviour at the critical moment. For example, people may forget to perform the intended behaviour (Einstein et al., 2003) or fail to initiate the response because they are unsure how to act when the moment presents itself (Gollwitzer & Sheeran, 2006). In other words, they experience a problem with enactment. Given this and preliminary evidence that some people are unfamiliar with how to respond with self‐compassion or tend to be self‐critical when they experience difficulties (e.g., Bayir & Lomas, 2016; Pauley & McPherson, 2010), we propose that difficulties enacting self‐compassionate responding may influence whether people respond with self‐compassion to lapses in pursuing their goals.

### Prototype willingness model

The TPB delineates the intentional and reasoned processes that influence behaviour. However, certain behaviours may also involve more reactive or heuristic processes (Sheeran et al., 2013). The PWM was developed to explain such behaviours and posits that there are two pathways that explain whether people will engage in a specific behaviour (Gibbons et al., 1998, 2009). The first path specifies the deliberative, reasoned processes that affect behaviour via intentions (as proposed by the TPB) and the second ‘social reaction’ path specifies the more reactive and spontaneous processes that affect behaviour via willingness. For example, although someone may not explicitly intend to respond with self‐compassion, they might be willing to do so should the opportunity arise. Like intentions, willingness is influenced by people's behavioural and normative beliefs about the behaviour. However, the PWM posits that willingness is also influenced by more reactive and spontaneous processes shaped in part by people's evaluations of the prototypical person that engages in the behaviour. For example, people high in PC may be less willing to respond with self‐compassion because they have negative evaluations of the prototypical person who responds with self‐compassion (e.g., they view them as weak). Considerable evidence has indicated that prototype evaluations and willingness predict a variety of behaviours even once the factors specified in the TPB are taken into account (Gerrard et al., 2008; Todd et al., 2016). Consistent with prior work suggesting that some people associate self‐compassion with self‐indulgence or prefer to respond with self‐criticism to difficulties (e.g., Bayir & Lomas, 2016; Robinson et al., 2016), we therefore propose that people's evaluations of the prototypical person who responds to difficulties with (i) high self‐compassion, (ii) self‐indulgence, or (iii) low self‐compassion may influence whether they are willing to respond with self‐compassion to a lapse in goal pursuit and, in turn, that willingness will predict self‐compassionate responding.

### The present study

This study proposes that social cognition models can be used to understand self‐compassionate responding. Specifically, we investigated whether an extended version of the TPB combined with the PWM can be used to predict (i) whether people will respond to lapses in goal pursuit with self‐compassion and (ii) why people with higher levels of PC may be less likely to respond with self‐compassion.

**FIGURE 1:**
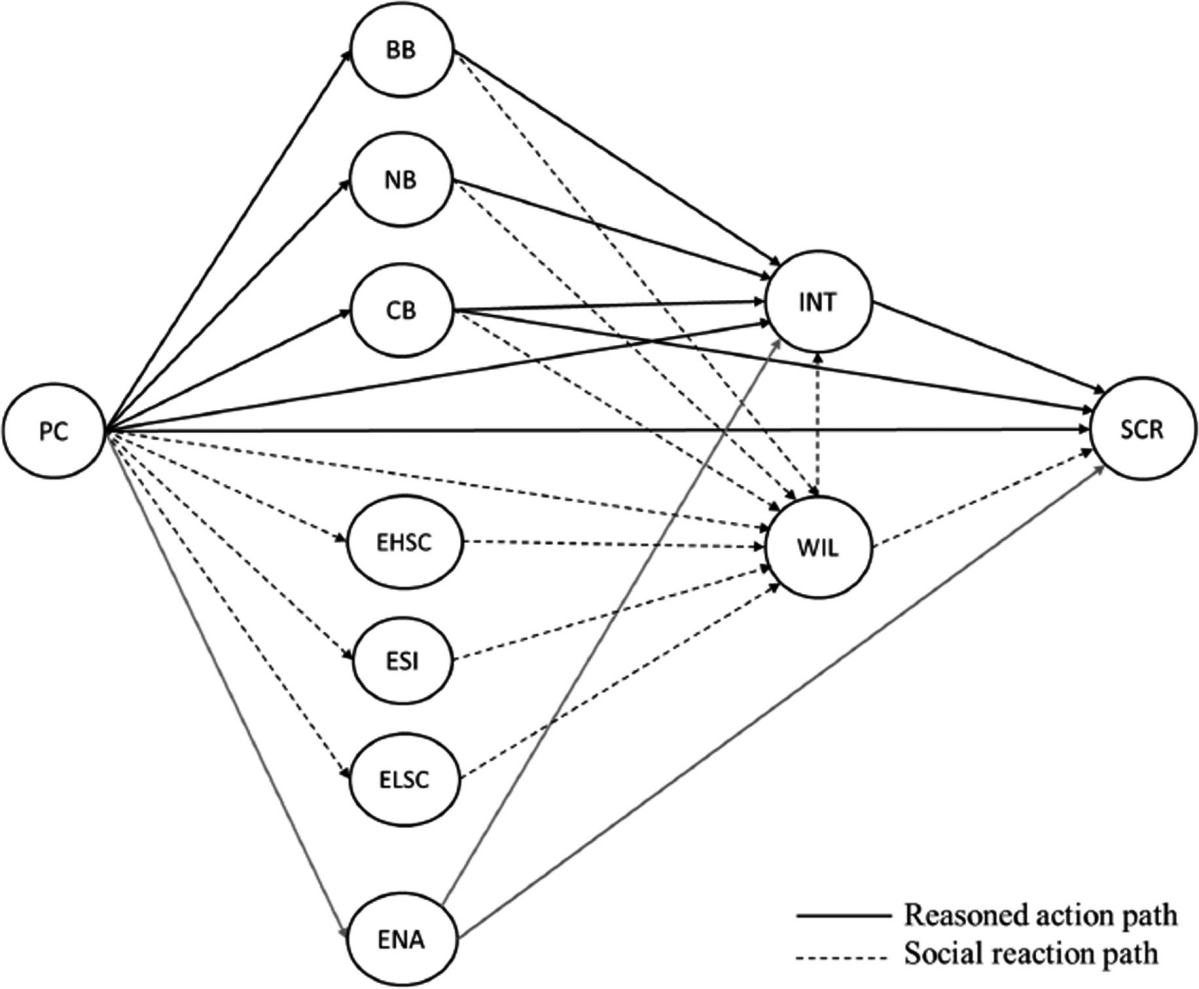
Proposed model for predicting self‐compassionate responding to a lapse in goal pursuit based on perfectionistic concerns, beliefs about responding with self‐compassion, and difficulties enacting self‐compassionate responding. *Note*. PC, perfectionistic concerns; BB, behavioural beliefs; NB, normative beliefs; CB, control beliefs; EHSC, prototype evaluations of responding with high self‐compassion; ESI, prototype evaluations of responding with self‐indulgence; ELSC, prototype evaluations of responding with low self‐compassion; ENA, difficulties enacting self‐compassionate responding; INT, intentions to respond with self‐compassion; WILL, willingness to respond with self‐compassion; SCR, self‐compassionate responding

To test our predictions, we conducted a study with a prospective design that was pre‐registered on the Open Science Framework (OSF; https://osf.io/huxqv). We predicted that positive behavioural and normative beliefs about self‐compassionate responding, positive evaluations of the prototypical person who responds with self‐compassion, and/or negative evaluations of the prototypical person who responds with self‐indulgence or low self‐compassion would be related to greater willingness to respond with self‐compassion to lapses in goal pursuit. In turn, we predicted that greater willingness, positive control beliefs, and less difficulties enacting self‐compassionate responding would be related to responding with self‐compassion to lapses. In addition, we predicted that higher levels of PC would be related to being less willing to respond with self‐compassion to lapses via negative behavioural and normative beliefs, negative prototype evaluations of responding with high self‐compassion, and/or positive evaluations of the prototypical person who responds with self‐indulgence or low self‐compassion. Additionally, we predicted that higher levels of PC would be related to being less likely to respond with self‐compassion to lapses via being less willing to respond with self‐compassion, negative control beliefs, and/or greater difficulties enacting self‐compassionate responding. Figure 1 depicts the proposed model with all the hypothesized effects and a full list of the hypotheses is available on OSF.

## METHOD

### Participants

To identify the sample size required to test the hypothesized effects, we conducted an a‐priori power analysis based on a Monte Carlo simulations approach for SEM (Wang & Rhemtulla, 2021; Wolf et al., 2013). We ran a series of simulations based on 2000 samples of different sizes with α = 0.02 (one‐tailed) to identify how many participants would provide at least 90% power to detect each of the hypothesized indirect effects of PC on willingness, intentions, and responding with self‐compassion, with the expected effect sizes ranging from 0.02 to 0.10 (more details about the power analysis can be found on OSF). These analyses recommended a target sample size of 1600 participants. Past research suggests that the percentage of participants who start but do not complete a survey in Prolific, an online recruitment tool, is 12% (Peer et al., 2017) and the drop‐out rate for a retrospective study with a follow‐up period of 2 weeks in this platform is 15% (Palan & Schitter, 2018). We therefore aimed to recruit at least 2061 participants to account for potential missing data and dropouts.

We recruited participants from Prolific. Anyone who was resident in the United Kingdom and self‐identified as struggling to achieve one or more personal goals was eligible to participate. We recruited 2116 participants, from whom we excluded those who did not provide thoughtful and complete answers (*N* = 37), failed to complete the attention check task (*N* = 17), or completed one of the surveys twice (*N* = 2). Following exclusions, 2060 participants (1247 women, 798 men, 14 other, 1 unknown) aged between 18 and 82 years (*M* = 35.86, *SD* = 13.58), predominantly Caucasians (83.4%) provided data at Time 1 (T1) and 1953 of these participants (94.8% response rate; 1178 women, 761 men, 13 other, 1 unknown) aged between 18 and 82 years (*M* = 36.10, *SD* = 13.62), predominantly Caucasians (82.9%) provided data at Time 2 (T2).^1^

### Procedure

Participants completed the study online and we did not restrict the amount of time that they had to respond to the writing tasks. At T1, we provided participants with information about the study as well as self‐compassion, and then asked them to write what they think that self‐compassion is about. Afterwards, we instructed participants to briefly describe a goal that they are currently struggling with. Participants’ responses to this latter question were coded using an established taxonomy for classifying personal goals (Reisz et al., 2013; see also Kaiser & Ozer, 1997). The majority of the participants wrote about health and fitness‐related goals (*n* = 746; 36.2%), followed by academic or occupational (*n* = 545; 26.5%), financial (*n* = 206; 10.0%), affect control (*n* = 197; 9.6%), organization (*n* = 103; 5.0%), social relationships (*n* = 82; 4.0%), independence (*n* = 37; 1.8%), and moral (*n* = 25; 1.2%), with some responses referring to more than one of the aforementioned goal types (*n* = 119; 5.7%). Participants then completed the demographic questions (i.e., reported their gender, age, ethnicity, and education level).

Next, we asked participants to complete a measure assessing trait levels of PC. Participants then completed measures assessing their behavioural, normative, and control beliefs with respect to responding to difficulties with self‐compassion, and how they evaluate the prototypical person who responds to difficulties with (i) high self‐compassion, (ii) self‐indulgence, or (iii) low self‐compassion. They also reported the extent to which they have encountered difficulties enacting self‐compassionate responding. Following these measures, we asked participants to complete an attention check as well as measures assessing social desirability and state levels of self‐compassion.

Afterwards, we instructed participants to think and write about a recent lapse that they experienced when pursuing their chosen goal and then indicate their feelings towards the lapse using a 5‐point faces scale (1 = very sad face to 5 = very happy face; *M* = 2.15, *SD* = 0.95). Following this, we asked participants to indicate their willingness and intentions to respond with self‐compassion to the lapse that they recalled. Next, we prompted participants to respond with self‐compassion to the lapse using the prompt developed by Sirois et al. (2019). Specifically, we instructed them to think about the lapse that they had written about and ‘write a couple of sentences expressing this kindness, understanding, and balanced perspective to yourself’. Following this writing task, we asked participants to complete the state self‐compassion scale again to assess any change in state self‐compassion before and after being prompted to respond with self‐compassion. Lastly, we asked participants to indicate their willingness and intentions to respond with self‐compassion to a lapse in their chosen goal or difficulties that they may face in the future.

At T2, participants were asked whether they remembered the goal that they described in the first questionnaire (yes or no). Participants who indicated that they did were asked whether they ‘experienced any lapse while working towards your goal over the last 2 weeks’ (yes or no) and, if they responded ‘yes’, to describe ‘the nature of this lapse and how you responded to it. What thoughts and feelings did you experience afterwards?’ Participants who could not remember their goal or stated that they had not experienced a lapse were instructed to ‘think about a difficulty or challenge that you have experienced over the last 2 weeks’ and to ‘describe the nature of this challenge and how you responded to it. What thoughts and feelings did you experience afterwards?’^2^ Finally, we asked participants to complete the measure of state self‐compassion again to assess the extent to which they responded with self‐compassion to the lapse in their goal or difficulty that they experienced.

### Measures

#### Perfectionistic concerns

Participants completed the short form of the Multidimensional Perfectionism Scale (Hewitt & Flett, 1991). This scale consists of three subscales, measured by five statements each, that assess Self‐Oriented Perfectionism, Other‐Oriented Perfectionism, and Socially Prescribed Perfectionism (e.g., ‘I feel that people are too demanding of me’). Participants indicated the extent to which they agreed with each statement (1 = *strongly disagree* to 7 = *strongly agree*). In line with theory and previous research (e.g., Sirois & Molnar, 2016; Stoeber, 2018; Stoeber et al., 2015), we used the Socially Prescribed Perfectionism subscale as a measure of perfectionistic concerns (PC).

#### Behavioural beliefs

Participants responded to items assessing affective and cognitive attitudes towards each of the three components of self‐compassion (self‐kindness, common humanity, and mindfulness). Nine items assessed affective attitudes (e.g., ‘For me, being kind and accepting toward myself when I experience difficulties is…’: Unpleasant to pleasant; enjoyable to unenjoyable) and nine items assessed cognitive attitudes (e.g., ‘For me, being kind and accepting toward myself when I experience difficulties is…’: Pointless to worthwhile; harmful to beneficial). Participants rated each adjective pair on a 7‐point unipolar scale.

#### Normative beliefs

Participants responded to items assessing injunctive, descriptive, personal, and cultural norms towards each of the three components of self‐compassion. Three items assessed injunctive norms (e.g., ‘People who are important to me think that I should be kind and accepting toward myself when I experience difficulties’), three items assessed descriptive norms (e.g., ‘People who are important to me see experiencing difficulties as part of being human’), three items assessed personal norms (e.g., ‘I personally feel that I should take a balanced view of the situation when they experience difficulties’), and three items assessed cultural norms (e.g., ‘People from my culture are kind and accepting toward themselves when they experience difficulties’). Participants indicated the extent to which they agreed with each statement (1 = *strongly disagree* to 6 = *strongly agree*).

#### Control beliefs

Participants responded to three items assessing control beliefs towards each of the three components of self‐compassion (e.g., ‘I am confident that I can see experiencing difficulties as part of being human’) and indicated the extent to which they agreed with each statement (1 = *strongly disagree* to 6 = *strongly agree*).

#### Prototype evaluations

Participants read three scenarios that provided examples of typical people who respond to difficulties with (i) high self‐compassion, (ii) self‐indulgence, and (iii) low self‐compassion, respectively. After reading each scenario, participants were asked to evaluate the person in the scenario using five pairs of adjectives on a 7‐point unipolar scale which, following Robinson et al. (2016), assessed performance‐related beliefs about self‐compassion (e.g., lazy to industrious; responsible to irresponsible).

#### Enactment difficulties

Participants indicated the extent to which they agreed with nine statements describing difficulties enacting self‐compassionate responding on a scale from 1 = *strongly disagree* to 6 = *strongly agree*. The stem ‘When I am experiencing difficulties…’ was followed by statements such as ‘I do not have the time to be kind and accepting towards myself’, ‘My critical inner voice gets in the way of seeing experiencing difficulties as part of being human’, ‘I struggle to take a balanced view of the situation’). The items were derived from previous studies exploring what impedes people from responding with self‐compassion (Bayir & Lomas, 2016; Campion & Glover, 2017).

#### Social desirability

Participants completed the Balanced Inventory of Desirable Responding (Hart et al., 2015). This scale consists of 16 items (e.g., ‘I never cover up my mistakes’) and participants indicated the extent to which they agreed with each statement (1 = *totally disagree* to 7 = *totally agree*).

#### Willingness and intentions

Participants responded to three items assessing their willingness to respond with self‐compassion and three items assessing their intentions to respond with self‐compassion to (i) a lapse in goal pursuit if prompted to respond with self‐compassion (e.g., ‘When I am asked to respond with self‐compassion to the lapse that I just described…’: ‘I intend to be kind and accepting toward myself’, ‘I am willing to be kind and accepting toward myself’) and (ii) a lapse or difficulty that they may face in the future (e.g. ‘If I experience a lapse in my goal or difficult time in the future, then…’: ‘I intend to take a balanced view of the situation’, ‘I am willing to take a balanced view of the situation’). Participants indicated the extent to which they agreed with each statement (1 = *strongly disagree* to 6 = *strongly agree*).

#### State self‐compassion

Participants completed the short form of the State Self‐Compassion Scale (SSCS‐S; Neff et al., 2021) before and after being prompted to respond to the recalled lapse with self‐compassion, as well as at T2. This scale consists of six items (e.g., ‘I’m giving myself the caring and tenderness I need’). Participants indicated the extent to which each statement is true for them (1 = *not at all true for me* to 5 = *very true for me*). When assessing self‐compassion at T2, we adapted the scale so that the items refer to the lapse. That is, the items were preceded by the stem ‘When you experienced the difficult time that you just described…’ and were phrased in the past tense (e.g., ‘I gave myself the caring and tenderness I needed’).

### Approach to analysis

We conducted structural equation modeling using AMOS 26.0 to test the model depicted in Figure 1. We specified two variants of the model predicting responding with self‐compassion to (i) a recalled lapse and (ii) a future lapse in goal pursuit, respectively. First, we conducted confirmatory factor analysis to evaluate the measurement part of the models. After establishing measurement models with acceptable fit, we added social desirability to the models as a covariate to examine whether it was associated with the factors in the models and thus should be included in the models. Next, we tested the full latent variable models to investigate the hypothesized effects between the factors in the models. The Maximum Likelihood Method was used to estimate the parameters of the models.

Each factor in the models was measured by multiple indicators. We specified each item of a measure as a single indicator of its corresponding factor. The only exceptions were the factors of behavioural and normative beliefs about responding with self‐compassion, as well as difficulties enacting self‐compassionate responding. Specifically, we computed and specified the means of the measures of affective attitudes (α = 0.88, coefficient *H* = 0.88, *M* = 4.48, *SD* = 1.07) and cognitive attitudes (α = 0.90, *H* = 0.90, *M* = 5.53, *SD* = 1.07) as the two indicators of behavioural beliefs and the means of the measures of injunctive norms (α = 0.88, *H* = 0.88, *M* = 4.94, *SD* = 0.90), descriptive norms (α = 0.85, *H* = 0.85, *M* = 4.27, *SD* = 0.94), personal norms (α = 0.87, *H* = 0.87, *M* = 5.09, *SD* = 0.89), and cultural norms (α = 0.87, *H* = 0.88, *M* = 3.78, *SD* = 1.06) as the four indicators of normative beliefs. Additionally, we randomly created three parcels, of three items each, as the three indicators of difficulties enacting self‐compassionate responding. We did so to achieve a more parsimonious model and deal with issues that lengthy unidimensional scales may cause (Kline, 2016; Yang et al., 2010).

We rejected the null hypothesis for the hypothesized main effects if *p* < .02 and for the indirect effects if the 98% confidence interval (5000 bootstrap samples) did not include zero. As recommended by Hu and Bentler (1999) and Kline (2016), we evaluated the fit of the models using two absolute fit indices: (i) the Chi‐square statistic with its degrees of freedom and (ii) the Root Mean Square Error of Approximation (RMSEA), and two incremental fit indices: (i) the Comparative Fit Index (CFI) and (ii) the Tucker‐Lewis Index (TLI). Because the Chi‐square statistic is sensitive to sample size, we mainly relied on RMSEA, CFI, and TLI which do not have a strong reliance on sample size (Byrne, 2010). Values above 0.90 for CFI and IFI and below 0.10 for RMSEA were taken to indicate acceptable model fit (Browne & Cudeck, 1993; Hu & Bentler, 1999). To compare the fit of two nested models, we computed the difference in the chi‐square values of the two models (Δχ^2^).

## RESULTS

The data from the study and the models that were tested are available on the OSF (https://osf.io/dav6x/?view_only=d74de0e216d345e197c4f3cffd81bdbd).

### Preliminary analyses

#### Missing data

The proportion of missing data was 0.3% and 0.1% for the first and second surveys, respectively (excluding participants who dropped out). To test whether there was a pattern to the missing data, we conducted the Little's MCAR test using SPSS 26.0. The test was significant, χ^2^ = 15,921.35, *df* = 14,649, *p* < .001, meaning that the data were not missing at random. To handle the missing data, we used the multiple imputation technique (Schafer & Graham, 2002) as recommended by Enders (2010).

#### Correlations

Table 1 shows the correlations between the factors in the models. The correlation between affective and cognitive attitudes was significant (*p* < .001) and positive (*r* = .68) indicating that they assess related, but distinct, behavioural beliefs about self‐compassionate responding. Similarly, the correlations among injunctive, descriptive, personal, and cultural norms were significant (*p* < .001) and positive (*r* = .15 to .42) indicating that they are related, but also discriminable, normative beliefs about self‐compassionate responding.

**TABLE 1:** Correlations between the factors associated with self‐compassionate responding to a recalled and future lapse

		1	2	3	4	5	6	7	8	9	10	11	12	13	14
1	PC	–													
2	Behavioural beliefs	−.28***	–												
3	Normative beliefs	−.23***	.70***	–											
4	Control beliefs	−.29***	.46***	.47***	–										
5	EHSC	−.12***	.46***	.45***	.17***	–									
6	ESI	−.12***	.23***	.20***	.13***	.36***	–								
7	ELSC	−.02	.08**	.06*	.03	.16***	.16***	–							
8	Enactment difficulties	.39***	−.31***	−.16***	−.60***	−.06**	−.05*	.04	–						
9	Intentions (recalled lapse)	−.18***	.47***	.49***	.48***	.24***	.13***	−.01	−.33***	–					
10	Willingness (recalled lapse)	−.19***	.51***	.55***	.51***	.27***	.16***	−.01	−.33***	.91***	–				
11	Time 1 self‐compassion	−.33***	.48***	.46***	.55***	.24***	.11***	−.01	−.47***	.52***	.58***	–			
12	Intentions (future lapse)	−.19***	.55***	.67***	.51***	.36***	.15***	.02	−.27***	–	–	–	–		
13	Willingness (future lapse)	−.18***	.53***	.66***	.49***	.35***	.15***	.02	−.25***	–	–	–	.97***	–	
14	Time 2 self‐compassion	−.27***	.33***	.25***	.45***	.16***	.08***	−.02	−.46***	–	–	–	.41***	.40***	–

Abbreviations: EHSC, prototype evaluations of responding with high self‐compassion; ELSC, prototype evaluations of responding with low self‐compassion; ESI, prototype evaluations of responding with self‐indulgence; PC, perfectionistic concerns.

*p < .02, **p < .01, ***p < .001.

### Measurement model

#### Initial models

The initial measurement models predicting self‐compassionate responding to a recalled lapse, χ^2^(847) = 5203.98, *p* < .001; CFI = 0.92, TLI = 0.91, RMSEA = 0.050, and future lapse, χ^2^(847) = 4965.65, *p* < .001; CFI = 0.92, TLI = 91, RMSEA = 0.050, provided an acceptable fit to the data. The modification indices revealed high covariances between the errors of the subscales assessing descriptive and cultural norms about self‐compassionate responding. These subscales measure the extent to which participants believe that other people (i.e., participants’ significant others and people from their culture) respond with self‐compassion and, as such, contain similar items (e.g., ‘People who are important to me/People from my culture see experiencing difficulties as part of being human’). We thus allowed their errors to correlate.

There were also very large correlations between the measures of willingness and intentions to respond with self‐compassion (Table 1), which meant that it was not possible to treat willingness and intentions to respond with self‐compassion separately in the models, despite evidence that willingness and intention can independently predict outcomes (e.g., Gibbons et al., 1998). Therefore, to investigate whether willingness or intentions best predicted responding with self‐compassion, we tested three models specifying (i) a unidirectional path from willingness to intentions, (ii) a unidirectional path from intentions to willingness, or (iii) a bidirectional path between intentions and willingness. The models showed that willingness was more strongly associated with self‐compassionate responses than intentions and that the social cognitions that we predicted would be associated with these constructs (e.g., behavioural, normative, and control beliefs) were more strongly associated with willingness than with intentions (see Figures S1–S3 in the supplementary analysis). Therefore, we decided to drop intention from the models and focus on willingness as a potential predictor of self‐compassionate responding.

#### Re‐specified models

The re‐specified measurement models predicting self‐compassionate responding to a recalled lapse, χ^2^(733) = 3632.44, *p* < .001; CFI = 0.94, TLI = 0.93, RMSEA = 0.044, and future lapse, χ^2^(733) = 3317.03, *p* < .001; CFI = 0.94, TLI = 0.93, RMSEA = 0.042, provided a good fit to the data. Table 2 shows the loadings of each item or subscale on their respective factors. All of the standardized factor loadings were above the recommended minimum of 0.50 (Ford et al., 1986) except for two subscales loading on normative beliefs (namely, items reflecting descriptive and cultural norms) and one item loading on state self‐compassion. However, all of the factor loadings were statistically significant (*p* < .001) and so we retained the re‐specified measurement models.

**TABLE 2:** Loadings of each item or subscale on their respective factors in the re‐specified (final) measurement models

		β
*Perfectionistic concerns (PC)*		
Item 1	Anything I do that is less than excellent will be seen as poor work by those around me	0.72***
Item 2	I feel that people are too demanding of me	0.57***
Item 3	Although they may not show it, other people get very upset with me when I slip up	0.63***
Item 4	My family expects me to be perfect	0.72***
Item 5	People expect nothing less than perfection from me	0.78***
*Behavioural beliefs about self‐compassionate responding*		
Subscale 1	Affective Attitudes	0.72***
Subscale 2	Cognitive Attitudes	0.94***
*Normative beliefs about self‐compassionate responding*		
Subscale 1	Injunctive Norms	0.55***
Subscale 2	Descriptive Norms	0.35***
Subscale 3	Cultural Norms	0.23***
Subscale 4	Personal Norms	0.78***
*Control beliefs about self‐compassionate responding*		
Item 1	I am confident that I can be kind & accepting toward myself when I experience difficulties	0.86***
Item 2	I am confident that I can see experiencing difficulties as part of being human	0.85***
Item 3	I am confident that I can take a balanced view of the situation when I experience difficulties	0.87***
*Prototype evaluations of responding with high self‐compassion*		
Item 1	Lazy vs. Industrious	0.70***
Item 2	A success vs. A failure	0.79***
Item 3	Responsible vs. Irresponsible	0.85***
Item 4	Competent vs. Incompetent	0.83***
Item 5	Favourable vs. Unfavourable	0.81***
*Prototype Evaluations of Responding with Self‐Indulgence*		
Item 1	Lazy vs. Industrious	0.74***
Item 2	A success vs. A failure	0.82***
Item 3	Responsible vs. Irresponsible	0.83***
Item 4	Competent vs. Incompetent	0.84***
Item 5	Favourable vs. Unfavourable	0.83***
*Prototype evaluations of responding with low self‐compassion*		
Item 1	Lazy vs. Industrious	0.63***
Item 2	A success vs. A failure	0.77***
Item 3	Responsible vs. Irresponsible	0.71***
Item 4	Competent vs. Incompetent	0.82***
Item 5	Favourable vs. Unfavourable	0.71***
*Difficulties enacting self‐compassionate responding*		
Parcel 1	e.g., When I am experiencing difficulties, I do not have time to take a balanced view of the situation	0.95***
Parcel 2	e.g., When I am experiencing difficulties, my critical inner voice gets in the way of being kind and accepting towards myself	0.89***
Parcel 3	e.g., When I am experiencing difficulties, I struggle to be kind and accepting towards myself	0.91***
*Willingness to respond with self‐compassion to a recalled lapse*		
Item 1	I am willing to be kind and accepting toward myself	0.89***
Item 2	I am willing to see experiencing the lapse as part of being human	0.91***
Item 3	I am willing to take a balanced view of the situation	0.89***
*Willingness to respond with self‐compassion to a future lapse*		
Item 1	I am willing to be kind and accepting toward myself	0.88***
Item 2	I am willing to see experiencing the lapse as part of being human	0.90***
Item 3	I am willing to take a balanced view of the situation	0.89***
*Time 1 Self‐compassion*		
Item 1	I’m giving myself the caring and tenderness I need	0.73***
Item 2	I’m obsessing and fixating on everything that's wrong	0.73***
Item 3	I’m remembering that there are lots of others in the world feeling like I am	0.47***
Item 4	I feel intolerant and impatient toward myself	0.71***
Item 5	I’m keeping things in perspective	0.72***
Item 6	I feel like I’m struggling more than others right now	0.64***
*Time 2 Self‐compassion*		
Item 1	I’m giving myself the caring and tenderness I need	0.72***
Item 2	I’m obsessing and fixating on everything that's wrong	0.69***
Item 3	I’m remembering that there are lots of others in the world feeling like I am	0.43***
Item 4	I feel intolerant and impatient toward myself	0.70***
Item 5	I’m keeping things in perspective	0.74***
Item 6	I feel like I’m struggling more than others right now	0.56***

Note

***p < .001.

Next, we examined the associations between social desirability and the factors in the models. Social desirability was associated with lower PC (β = −0.13), less difficulties enacting self‐compassionate responding (β = −0.42), more positive behavioural (β = 0.23), normative (β = 0.14), and control beliefs (β = 0.42) about responding with self‐compassion, more positive evaluations of the prototypical person who responds with high self‐compassion (β = 0.08), greater willingness to respond with self‐compassion to a recalled lapse (β = 0.25) and future lapse (β = 0.24), as well as greater state self‐compassion at T1 (β = 0.34) and T2 (β = 0.33). All of these associations were significant at *p* < .001. We therefore added them to the structural models to adjust for the influence of social desirability.

### Structural models

#### Initial models

The initial structural models predicting self‐compassionate responding to a recalled lapse, χ^2^(833) = 6756.47, *p* < .001; CFI = 0.88, TLI = 0.87, RMSEA = 0.059, and future lapse, χ^2^(794) = 5499.49, *p* < .001; CFI = 0.89, TLI = 0.89, RMSEA = 0.055, provided a relatively poor fit to the data.

#### Re‐specified models

After inspecting the modification indices, we re‐specified the models to include a regression path from control beliefs about responding with self‐compassion to willingness to respond with self‐compassion to a past or future lapse. This is consistent with evidence that people's beliefs about whether they have control over the target behaviour may have a direct effect on willingness to perform that behaviour (e.g., Rivis et al., 2011). Additionally, we added two residual covariances between behavioural beliefs and evaluations of the prototypical person who responds with high self‐compassion, as well as between control beliefs and difficulties enacting self‐compassionate responding. The re‐specified structural models predicting self‐compassionate responding to a recalled lapse, χ^2^(830) = 5986.95, *p* < .001; CFI = 0.90, TLI = 0.89, RMSEA = 0.055, and future lapse, χ^2^(791) = 4724.64, *p* < .001; CFI = 0.91, TLI = 0.90, RMSEA = 0.050, provided an acceptable fit to the data.

#### Alternative models

**FIGURE 2:**
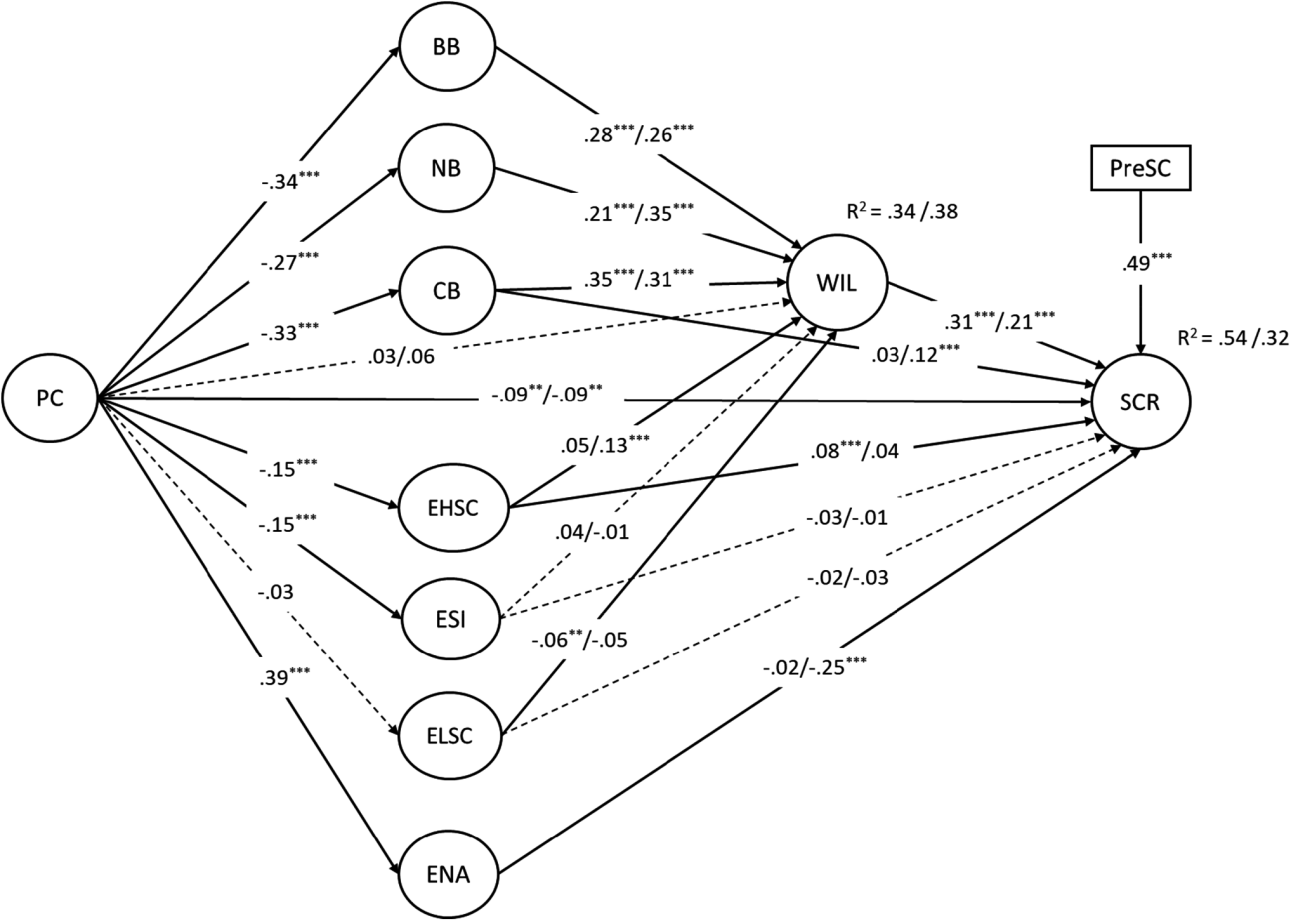
Final model predicting self‐compassionate responding to a recalled and future lapse in goal pursuit based on perfectionistic concerns, beliefs about responding with self‐compassion, and difficulties enacting self‐compassionate responding. Path coefficients are standardized and reported separately for the models predicting self‐compassionate responding to a recalled/future lapse. The measurement part of the model, paths representing associations with social desirability, and error terms were estimated but omitted from the figure for clarity. Dashed paths indicate non‐significant associations. *Note*. **p < .01, ***p < .001. PC, perfectionistic concerns; BB, behavioural beliefs; NB, normative beliefs; CB, control beliefs; EHSC, prototype evaluations of responding with high self‐compassion; ESI, prototype evaluations of responding with self‐indulgence; ELSC, prototype evaluations of responding with low self‐compassion; ENA, difficulties enacting self‐compassionate responding; WIL, willingness to respond with self‐compassion; SCR, self‐compassionate responding; PreSC, state self‐compassion before the self‐compassionate responding prompt

Next, we compared the re‐specified structural models with an alternative structural model that included direct paths from evaluations of the prototypical person who responds with (i) high self‐compassion, (ii) self‐indulgence, or (iii) low self‐compassion to state self‐compassion at T1 and T2. This is consistent with evidence that people's evaluations of the prototypical person who engages in a target behaviour may have a direct effect on that behaviour (e.g., Rivis & Sheeran, 2013). These alternative models predicting self‐compassionate responding to a recalled lapse, χ^2^(827) = 5972.76, *p* < .001; CFI = 0.90, TLI = 0.89, RMSEA = 0.055, or future lapse, χ^2^(788) = 4721.55, *p* < .001; CFI = 0.91, TLI = 0.90, RMSEA = 0.051, also provided an acceptable fit to the data. The alternative model predicting self‐compassionate responding to a recalled lapse provided a better fit to the data than the re‐specified model, Δχ^2^ (3) = 14.19, *p* < .01, but the model predicting self‐compassionate responding to a future lapse did not, Δχ^2^ (3) = 3.09, *p* = .378. We therefore retained the alternative models. Figure 2 shows the final models depicting the regression paths.

#### Predictors of willingness to respond with self‐compassion

Positive behavioural, normative, and control beliefs about responding with self‐compassion were associated with being more willing to respond with self‐compassion to both a recalled lapse and future lapse. Additionally, negative evaluations of the prototypical person who responds with low self‐compassion and positive evaluations of the prototypical person who responds with high self‐compassion had weaker, but still statistically significant, associations with being more willing to respond with self‐compassion to a recalled lapse and future lapse, respectively.

#### Predictors of self‐compassionate responding

Willingness to respond with self‐compassion to a recalled lapse and future lapse was associated with greater state self‐compassion at T1 and at T2, respectively. Additionally, difficulties enacting self‐compassionate responding were associated with lower self‐compassion at T2. Furthermore, positive evaluations of the prototypical person who responds with high self‐compassion and positive control beliefs about responding with self‐compassion had weaker, but still statistically significant, associations with greater state self‐compassion at T1 and at T2, respectively.

#### Associations with PC

PC was associated with more negative behavioural, normative, and control beliefs about responding with self‐compassion, as well as more difficulties enacting self‐compassionate responding. Additionally, PC had weaker, but still statistically significant, associations with more negative evaluations of the prototypical person who responds with high self‐compassion or self‐indulgence.

Next, we examined the indirect effects of PC on willingness to respond with self‐compassion and self‐compassionate responding (Table 3). The indirect effects of PC on willingness through behavioural, normative, and control beliefs about responding with self‐compassion were significant and negative; as was the indirect effect of PC on willingness to respond with self‐compassion to a future lapse through evaluations of the prototypical person who responds with high self‐compassion. Regarding self‐compassionate responding, the indirect effects of PC on state self‐compassion at T2 through control beliefs about responding with self‐compassion and difficulties enacting self‐compassionate responding were statistically significant; as was the indirect effect of PC on state self‐compassion at T1 through evaluations of the prototypical person who responds with high self‐compassion.

**TABLE 3:** Indirect effects of perfectionistic concerns on willingness to respond with self‐compassion and responding with self‐compassion to a recalled lapse and future lapse in goal pursuit

Indirect effects tested	Recalled lapse		Future lapse	
	*ab*	98% CI	*ab*	98% CI
PC ⇒ Behavioural Beliefs ⇒ Willingness	−0.085	−0.120/−0.059	−0.053	−0.079/−0.033
PC ⇒ Normative Beliefs ⇒ Willingness	−0.052	−0.087/−0.026	−0.061	−0.101/−0.033
PC ⇒ Control Beliefs ⇒ Willingness	−0.101	−0.133/−0.074	−0.057	−0.078/−0.039
PC ⇒ EHSC ⇒ Willingness	−0.006	−0.018/0.002	−0.012	−0.023/−0.005
PC ⇒ ESI ⇒ Willingness	−0.005	−0.013/0.002	0.001	−0.005/0.008
PC ⇒ ELSC ⇒ Willingness	0.002	−0.001/0.007	0.001	−0.001/0.006
PC ⇒ Control Beliefs ⇒ Self‐Compassion	−0.005	−0.022/0.011	−0.021	−0.040/−0.005
PC ⇒ EHSC ⇒ Self‐Compassion	−0.007	−0.014/−0.002	−0.003	−0.011/0.002
PC ⇒ ESI ⇒ Self‐Compassion	0.003	−0.001/0.008	0.001	−0.005/0.008
PC ⇒ ELSC ⇒ Self‐Compassion	0.001	−0.001/0.003	0.001	−0.001/0.005
PC ⇒ Enactment Difficulties ⇒ Self‐Compassion	−0.004	−0.018/0.010	−0.059	−0.080/−0.040
PC ⇒ Willingness ⇒ Self‐Compassion	0.005	−0.006/0.017	0.009	−0.001/0.021

Abbreviations: EHSC, prototype evaluations of responding with high self‐compassion; ELSC, prototype evaluations of responding with low self‐compassion; ESI, prototype evaluations of responding with self‐indulgence; PC, perfectionistic concerns.

## DISCUSSION

The findings of the present research support the idea that social cognition models can be used to understand whether people respond with self‐compassion and why. Specifically, the findings showed that an extended version of the TPB combined with aspects of the PWM predicted whether people were willing to respond to lapses in goal pursuit with self‐compassion. Participants who had more positive behavioural, normative, and control beliefs with respect to responding with self‐compassion, and more positive evaluations of the prototypical person who responds with high (instead of low) self‐compassion were more willing to respond with self‐compassion. In turn, participants who were more willing to respond with self‐compassion, had less difficulties enacting self‐compassionate responding, more positive control beliefs about responding with self‐compassion, and more positive evaluations of the prototypical person who responds with high self‐compassion were more likely to respond with self‐compassion.

These findings support previous preliminary evidence that holding negative views towards being self‐compassionate can mean that some people struggle to be self‐compassionate in times of difficulty (e.g., Chwyl et al., 2020; Robinson et al., 2016). Importantly, however, they extend this work to delineate the nature of these negative views and demonstrate their distinctive impact on responding with self‐compassion. For example, people who lack confidence in their ability to be self‐compassionate are more likely to struggle to respond with self‐compassion, even if they believe that doing so is beneficial and pleasant. Additionally, the present findings extend prior work by showing that, even if people have positive views towards being self‐compassionate and are willing to respond with self‐compassion, they may still struggle to do so, because they have difficulty enacting the self‐compassionate response. This finding supports the idea that motivation alone is often insufficient to drive behaviour (e.g., Sheeran & Webb, 2016; Webb & Sheeran, 2006), and points to the need to consider volitional factors that influence whether people translate willingness into action, such as whether people remember to perform the intended behaviour or know how to act at the critical moment (Gollwitzer & Sheeran, 2006; Sheeran & Webb, 2012).

This study also demonstrated that social cognitions specified in the TPB and the PWM can explain why people with higher levels of PC may be less likely to respond with self‐compassion—again providing evidence that social cognition models can be used to understand self‐compassionate responding. Specifically, participants high in PC were less willing to respond with self‐compassion to a lapse because they had more negative behavioural, normative, and control beliefs about responding with self‐compassion, as well as more negative evaluations of the prototypical person who responds with high self‐compassion. Additionally, participants high in PC were less likely to respond with self‐compassion to a lapse, because they had more negative control beliefs about responding with self‐compassion, more difficulties enacting self‐compassionate responding, and evaluated the prototypical person who responds with high self‐compassion more negatively.

These findings support previous evidence that people high in PC are likely to struggle to be self‐compassionate (e.g., Stoeber et al., 2020; Tobin & Dunkley, 2021). Crucially, however, this study extends prior work by showing that negative beliefs about self‐compassion can explain why people high in PC struggle to respond with self‐compassion. Of course, such evidence also raises the question of why people high in PC hold negative beliefs about self‐compassion. Prior work has shown that people high in PC believe that their self‐worth depends on the extent to which they work towards meeting their high standards (Flett et al., 2003; Sturman et al., 2009) and thus strive to achieve their goals to consider themselves worthwhile (Flett & Hewitt, 2006; Spence & Robbins, 1992). We suspect that this contingent self‐worth explains why people high in PC hold negative beliefs about self‐compassionate responding, based on evidence that some people see self‐compassionate responding as being counterproductive (e.g., Kelly et al., 2021; Robinson et al., 2016).^3^ The finding that PC is related to being less likely to respond with self‐compassion via the beliefs specified in social cognition models may also provide the basis for investigating additional personality traits that may impede responding with self‐compassion. For example, habitual worrying and depression have both been found associated with greater levels of PC and, similarly to PC, with stronger self‐criticism (e.g., Santanello & Gardner, 2007; Stöber & Joormann, 2001).

### Practical implications

In addition to developing our understanding of the factors that are associated with self‐compassionate responding, using social cognition models to understand self‐compassion has important practical and therapeutic implications. For example, there has been enduring interest in developing interventions focused on cultivating a self‐compassionate stance. Meta‐analyses have demonstrated that self‐compassion interventions typically lead to a medium‐sized increase in self‐compassion immediately after participants complete the intervention, but only a small sized increase at later follow‐up periods (Ferrari et al., 2019; Kirby et al., 2017). These findings suggest that some people who could potentially benefit from receiving a self‐compassion intervention are unwilling or unable to adopt a self‐compassionate stance. Clinical conceptualizations of self‐compassion have emphasized how fears of compassion can be a barrier to responding with self‐compassion (Gilbert et al., 2011) and interventions such as Compassion Focused Therapy (Gilbert, 2014), which are designed to target such fears, have been shown to be effective for addressing this barrier in clinically vulnerable populations (Craig et al., 2020). Our research, using a social‐cognitive approach, suggests that part of this struggle might also be explained by negative beliefs about being self‐compassionate, difficulties enacting self‐compassionate responding, and/or specific personality traits such as PC. Consequently, self‐compassion interventions that are delivered outside of a clinical context should incorporate strategies designed specifically for addressing the identified challenges, such as volitional strategies (see Gollwitzer, 1999, 2014), to increase the likelihood that people can harvest the benefits of self‐compassion on goal pursuit and well‐being.

### Limitations and future directions

The findings of this study need to be interpreted in the context of some limitations to the methodology. First, the correlational, prospective design precludes causal inferences, particularly with respect to associations between the factors that were measured at the same point in time (i.e., cross‐sectionally). Having said this, the presumed temporal precedence of the link between PC and beliefs about self‐compassion is consistent with previous research showing that perfectionism leads to personal beliefs and attitudes (e.g., Boone et al., 2012, 2014). Relatedly, the direction of the proposed and tested associations between the social cognitions and self‐compassionate responding is consistent with the TPB and PWM (Ajzen, 1991; Gibbons et al., 1998) as well as studies using longitudinal designs (e.g., Dohnke et al., 2015; Matterne et al., 2011). Nonetheless, experimental research manipulating the proposed social cognitions (for examples, see Rivis & Sheeran, 2013; Sniehotta, 2009) or PC (see Boone et al., 2012) and testing the effect of so doing on self‐compassionate responding would be useful to confirm the temporal precedence proposed in the current research.

A second limitation is that we assessed the factors in the models using self‐report measures, which may be biased by social desirability concerns. We attempted to minimize this bias by assessing participants’ levels of social desirability and controlling statistically for its associations with the factors in the models. Additionally, our measure of self‐compassionate responding to a future lapse in goal pursuit required participants to recall how they responded to a lapse that may have occurred a few weeks ago and thus could be subject to recall bias. Although we found similar results when testing the model predicting self‐compassionate responding after participants were prompted to respond with self‐compassion to a recalled lapse, future research should consider alternative methods of measuring beliefs about self‐compassion (e.g., implicit measures; see Howell & Ratliff, 2017; Vantomme et al., 2005) and alternative measures of self‐compassionate responding in day‐to‐day life, such as daily diary methods.

Finally, the present research found a very large correlation between measures of willingness and intentions to respond with self‐compassion, despite evidence that willingness and intention are conceptually distinct (in the sense that willingness accounts for how people might respond should an appropriate opportunity arise, while intentions reflect what people plan to do at a given moment in time) and can independently predict outcomes (e.g., Gibbons et al., 1998). One explanation for the strong correlation might be that the present research asked participants about their intentions to respond with self‐compassion in the context of an opportunity to do so (i.e., they were taking part in a study that invited them to reflect on a recent lapse or difficulty and the respective measures explicitly stated this opportunity—‘When I am asked to respond with self‐compassion to the lapse that I just described…’, and ‘If I experience a lapse in my goal or difficult time in the future, then…’). Asking about intentions in this context may have meant that willingness and intentions were conceptually similar. Subsequent research might consider asking participants to report their intentions before being offered a specific opportunity to enact those intentions.

## CONCLUSION

The present research provided evidence that social cognition models can be used to understand self‐compassionate responding by showing that factors specified in the TPB and PWM explain why certain people, such as those high in PC, struggle to respond with self‐compassion when they experience a lapse in goal pursuit. This evidence paves the way for developing strategies to address the challenges of being self‐compassionate and thus help people to successfully pursue their goals.

## CONFLICT OF INTEREST

All authors declare no conflict of interest.

## AUTHOR CONTRIBUTION

**Marios Biskas:** Conceptualization; Formal analysis; Investigation; Methodology; Project administration; Visualization; Writing—original draft; Writing—review & editing. **Fuschia Sirois:** Conceptualization; Funding acquisition; Investigation; Methodology; Project administration; Resources; Supervision; Validation; Writing—review & editing. **Thomas Webb:** Conceptualization; Funding acquisition; Investigation; Methodology; Project administration; Resources; Supervision; Validation; Writing—review & editing.

### OPEN RESEARCH BADGES

This article has been awarded Open Materials, Open Data, Preregistered Research Designs Badges. All materials and data are publicly accessible via the Open Science Framework at https://osf.io/dav6x/?view_only=d74de0e216d345e197c4f3cffd81bdbd and www.osf.io/huxqv.

## Data Availability

The data that support the findings of this research are openly available on the Open Science Framework at https://osf.io/dav6x/?view_only=d74de0e216d345e197c4f3cffd81bdbd, Biskas, M., Sirois, F., & Webb, T. L. (2021, June 15). Using Social Cognition Models to Understand Responding with Self‐Compassion.

## Footnotes

^1^
We also assessed participants’ education level. Regarding participants who provided data at T1, 3.7% of them received some high school education, 10.6% were high school graduates, 24.0% received some university education, 39.9% were university graduates, 3.6% received some postgraduate education, 17.9% were postgraduates, and 0.3% did not provide information about their education level. Regarding participants who provided data at T2, 3.6% of participants received some high school education, 10.8% were high school graduates, 23.6% received some university education, 40.2% were university graduates, 3.7% received some postgraduate education, 17.8% were postgraduates, and 0.3% did not provide information about their education level.

^2^

*N* = 1137 participants reported experiencing a lapse in their chosen goal during the follow‐up period and *N* = 816 participants could not remember their chosen goal or did not report experiencing a lapse during the follow‐up period and, thus, were instructed to write about a difficulty that they experienced instead. Figure S4 of the supplementary analysis reports the analyses separately for these two groups of participants.

^3^
The present research provided preliminary evidence that contingent self‐worth may explain why people high in PC hold certain negative beliefs about being self‐compassionate (namely, behavioural and control beliefs) and have difficulties enacting self‐compassionate responding (see Figure S5 and Table S1 in the supplementary analysis).
