# Supplementary material for: Using social cognition models to understand why people, such as perfectionists, struggle to respond with self‐compassion
Source: Br J Soc Psychol. 2022 Mar 9;61(4):1160–82. doi: 10.1111/bjso.12531 (PMC9790291; doi:10.1111/bjso.12531)
Supplement: Supplementary file 1 [file BJSO-61-1160-s001.docx]

**
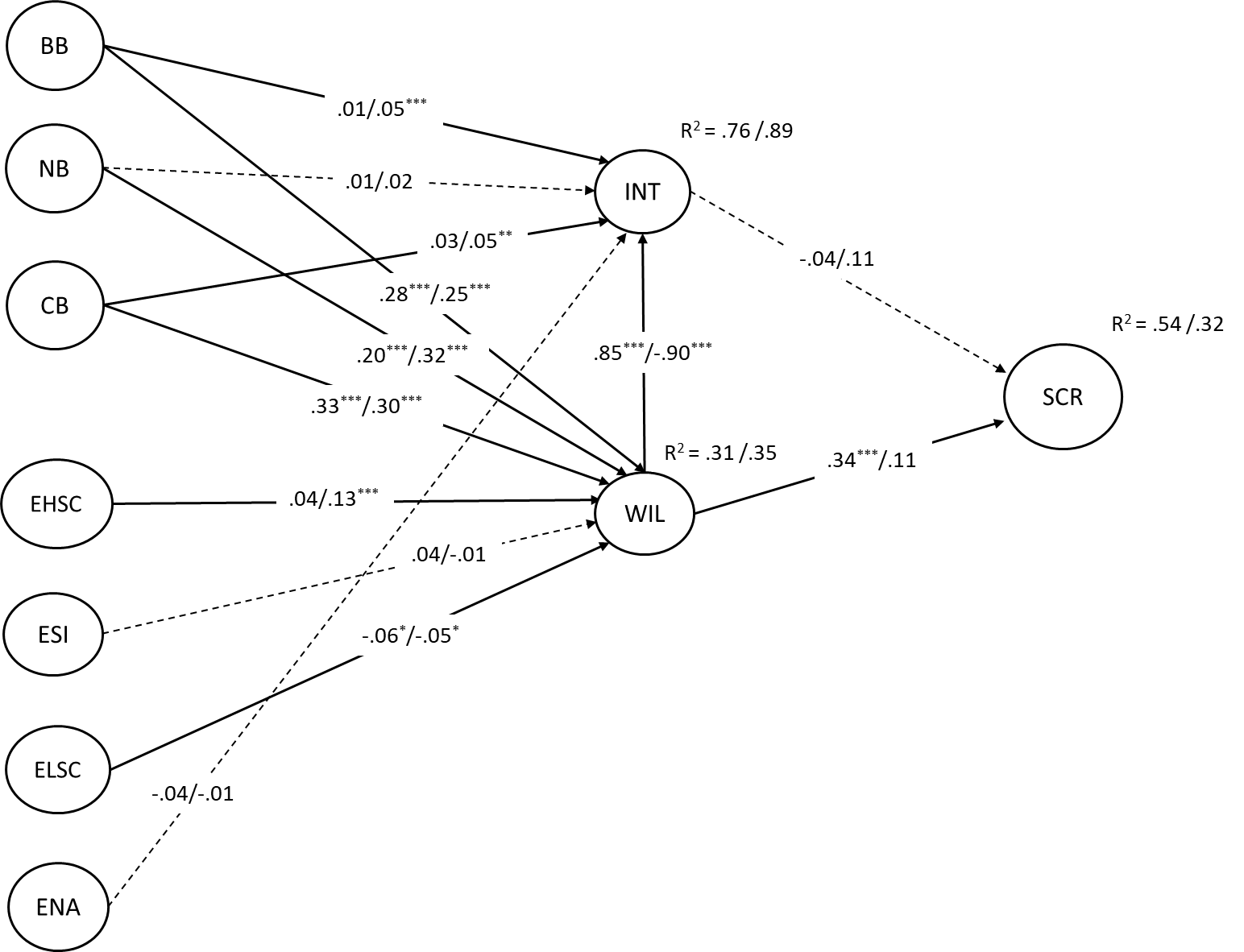
Supplementary Analysis**

**Figure S1.** Model predicting self-compassionate responding to a recalled and future lapse in goal pursuit with willingness predicting intentions. Path coefficients are standardized and reported separately for the models predicting self-compassionate responding to a recalled / future lapse. The measurement part of the model, paths representing associations with social desirability, and error terms were estimated but omitted from the figure for clarity. Dashed paths indicate non-significant associations.

*Note*. ^*^*p* < .02, ^**^*p* < .01, ^***^*p* < .001. PC = perfectionistic concerns, BB = behavioural beliefs, NB = normative beliefs, CB = control beliefs, EHSC = prototype evaluations of responding with high self-compassion, ESI = prototype evaluations of responding with self-indulgence, ELSC = prototype evaluations of responding with low self-compassion, ENA = difficulties enacting self-compassionate responding, INT = intentions to respond with self-compassion to a lapse, WIL = willingness to respond with self-compassion to a lapse, SCR = self-compassionate responding to a lapse, PreSC = state self-compassion before the self-compassionate responding prompt.


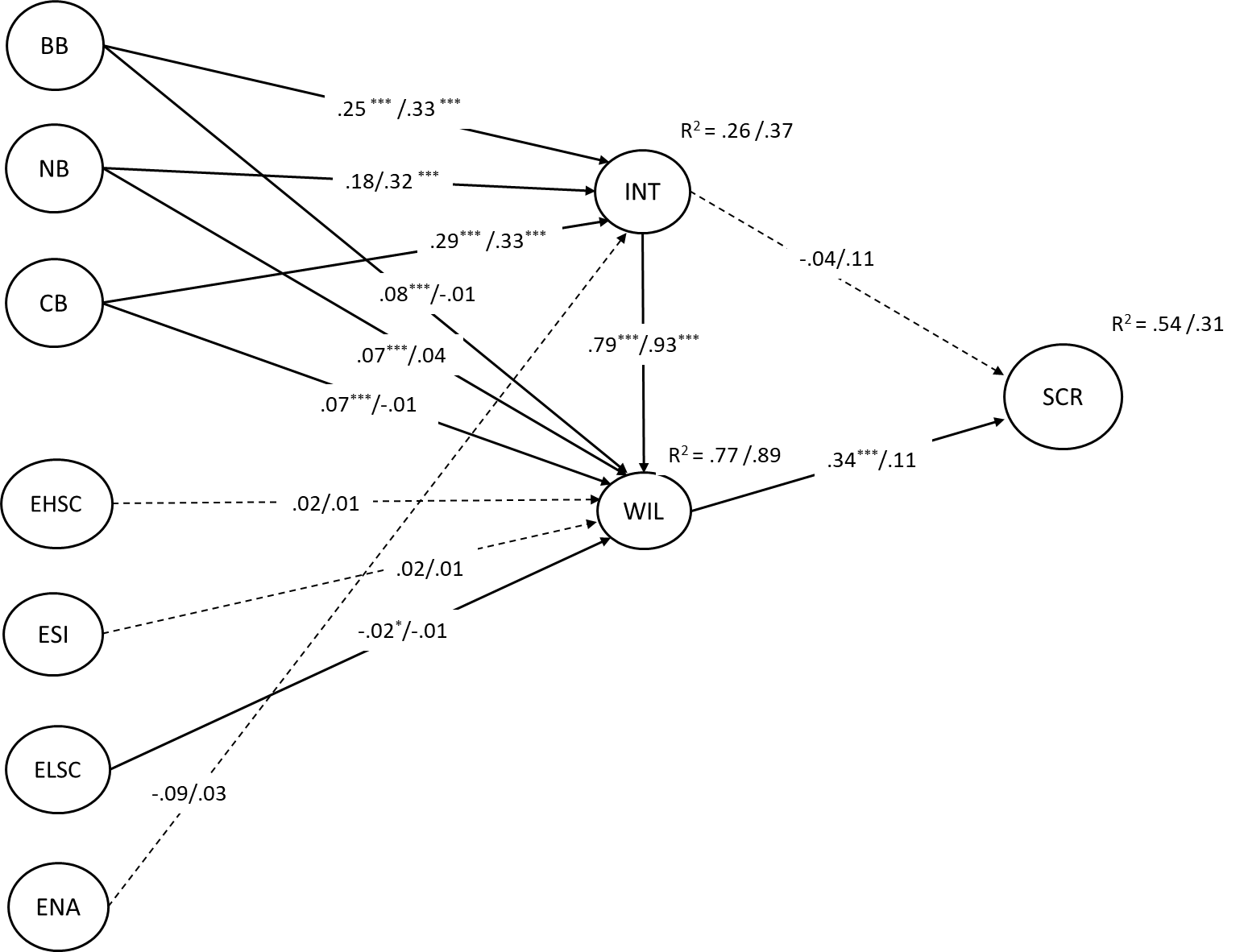


**Figure S2.** Model predicting self-compassionate responding to a recalled and future lapse in goal pursuit with intentions predicting willingness. Path coefficients are standardized and reported separately for the models predicting self-compassionate responding to a recalled / future lapse. The measurement part of the model, paths representing associations with social desirability, and error terms were estimated but omitted from the figure for clarity. Dashed paths indicate non-significant associations.

*Note*. ^*^*p* < .02, ^***^*p* < .001. PC = perfectionistic concerns, BB = behavioural beliefs, NB = normative beliefs, CB = control beliefs, EHSC = prototype evaluations of responding with high self-compassion, ESI = prototype evaluations of responding with self-indulgence, ELSC = prototype evaluations of responding with low self-compassion, ENA = difficulties enacting self-compassionate responding, INT = intentions to respond with self-compassion to a lapse, WIL = willingness to respond with self-compassion to a lapse, SCR = self-compassionate responding to a lapse, PreSC = state self-compassion before the self-compassionate responding prompt.


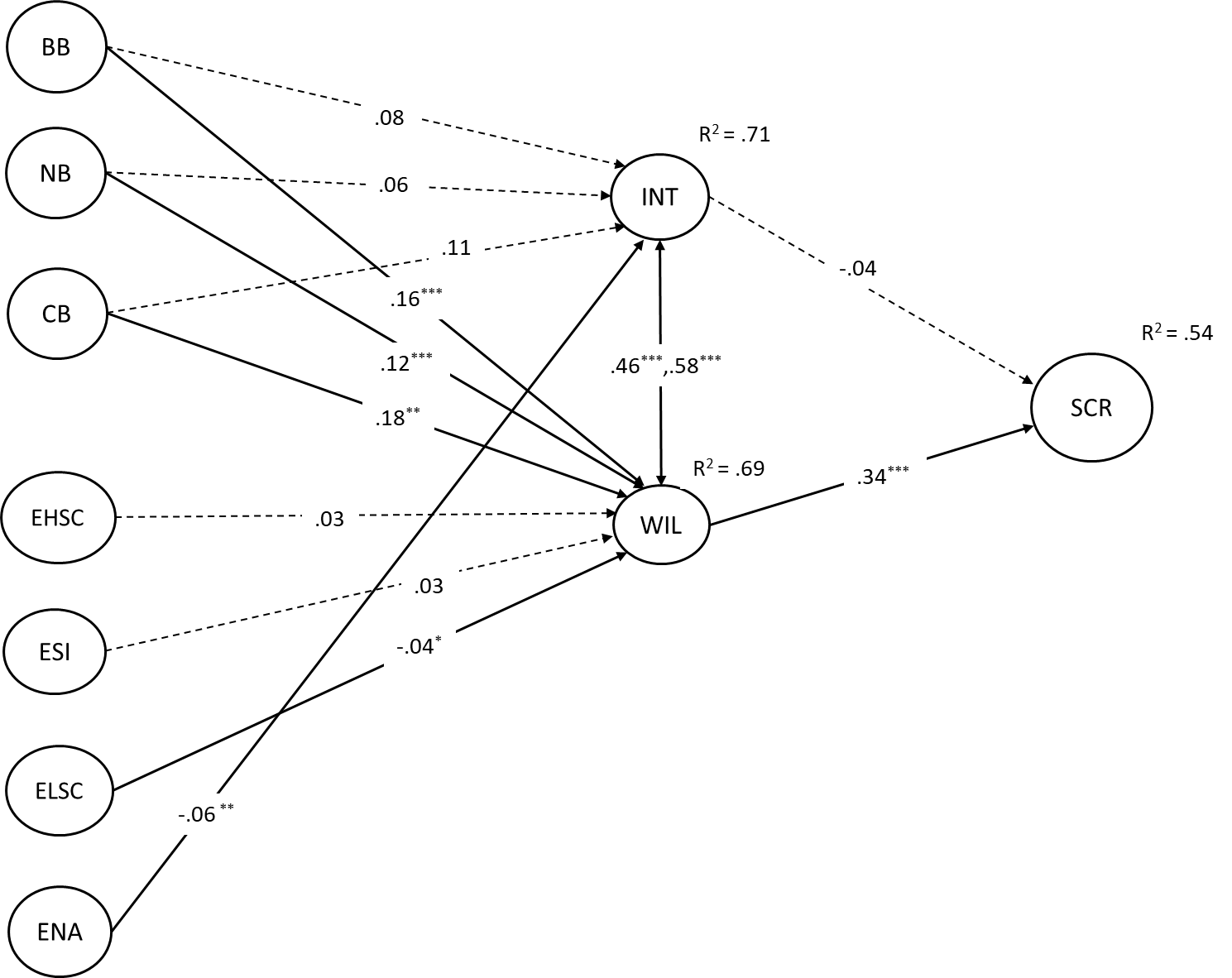


**Figure S3.** Model predicting self-compassionate responding to a recalled lapse in goal pursuit with intentions predicting willingness and willingness predicting intentions (bidirectional path). Path coefficients are standardized. The measurement part of the model, paths representing associations with social desirability, and error terms were estimated but omitted from the figure for clarity. Dashed paths indicate non-significant associations.

*Note*. ^*^*p* < .02, ^**^*p* < .01, ^***^*p* < .001. PC = perfectionistic concerns, BB = behavioural beliefs, NB = normative beliefs, CB = control beliefs, EHSC = prototype evaluations of responding with high self-compassion, ESI = prototype evaluations of responding with self-indulgence, ELSC = prototype evaluations of responding with low self-compassion, ENA = difficulties enacting self-compassionate responding, INT = intentions to respond with self-compassion to a lapse, WIL = willingness to respond with self-compassion to a lapse, SCR = self-compassionate responding to a lapse, PreSC = state self-compassion before the self-compassionate responding prompt.


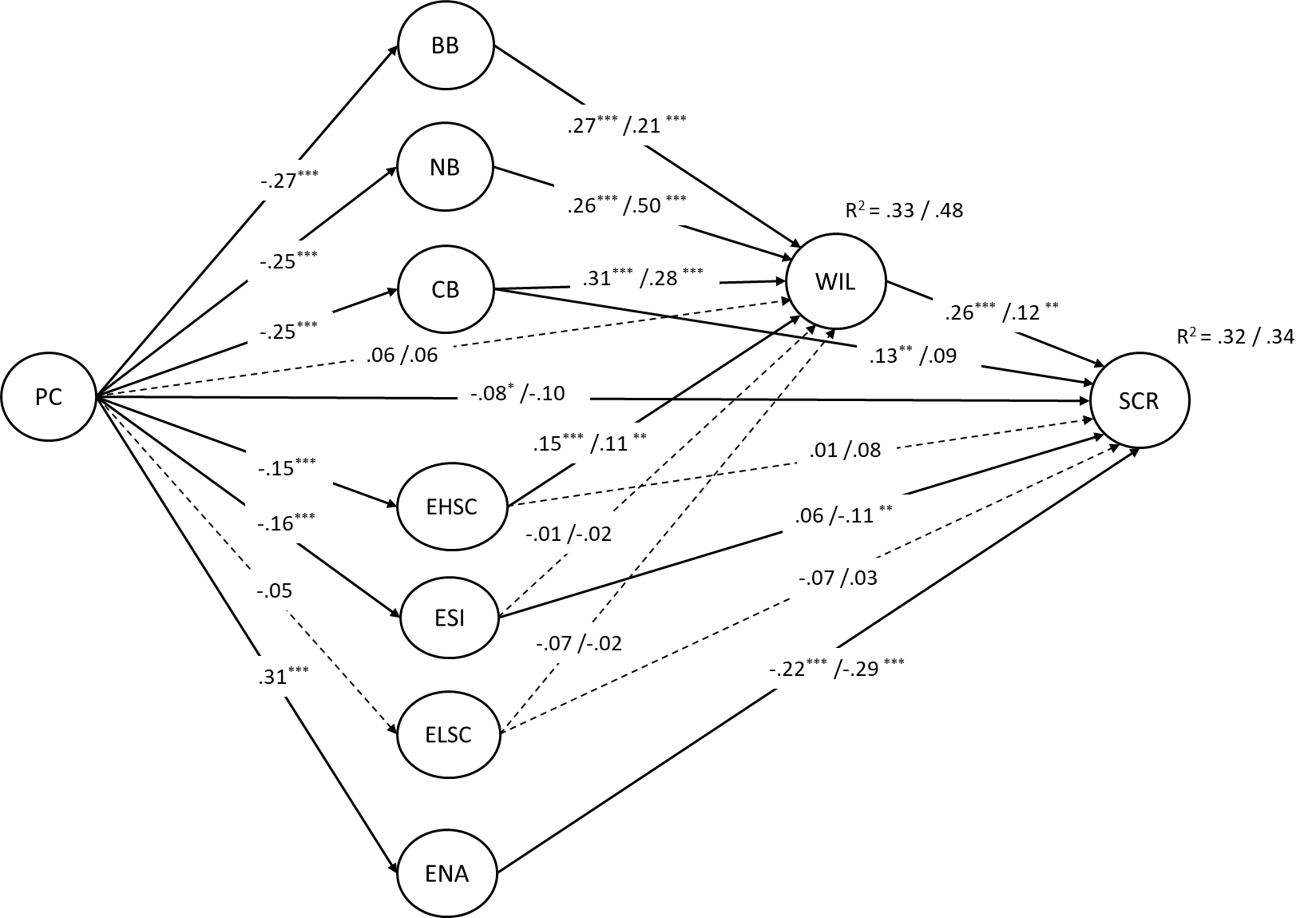


**Figure S4.** Model predicting self-compassionate responding to a future lapse in goal pursuit. Path coefficients are standardized and reported separately for the models including only participants who experienced a goal lapse (*n* = 1,137) / participants who did not remember their chosen goal or did not experience a goal lapse (*n* = 816). The measurement part of the model, paths representing associations with social desirability, and error terms were estimated but omitted from the figure for clarity. Dashed paths indicate non-significant associations.

*Note*. ^*^*p* < .02, ^**^*p* < .01, ^***^*p* < .001. PC = perfectionistic concerns, BB = behavioural beliefs, NB = normative beliefs, CB = control beliefs, EHSC = prototype evaluations of responding with high self-compassion, ESI = prototype evaluations of responding with self-indulgence, ELSC = prototype evaluations of responding with low self-compassion, ENA = difficulties enacting self-compassionate responding, WIL = willingness to respond with self-compassion to a lapse, SCR = self-compassionate responding to a lapse.


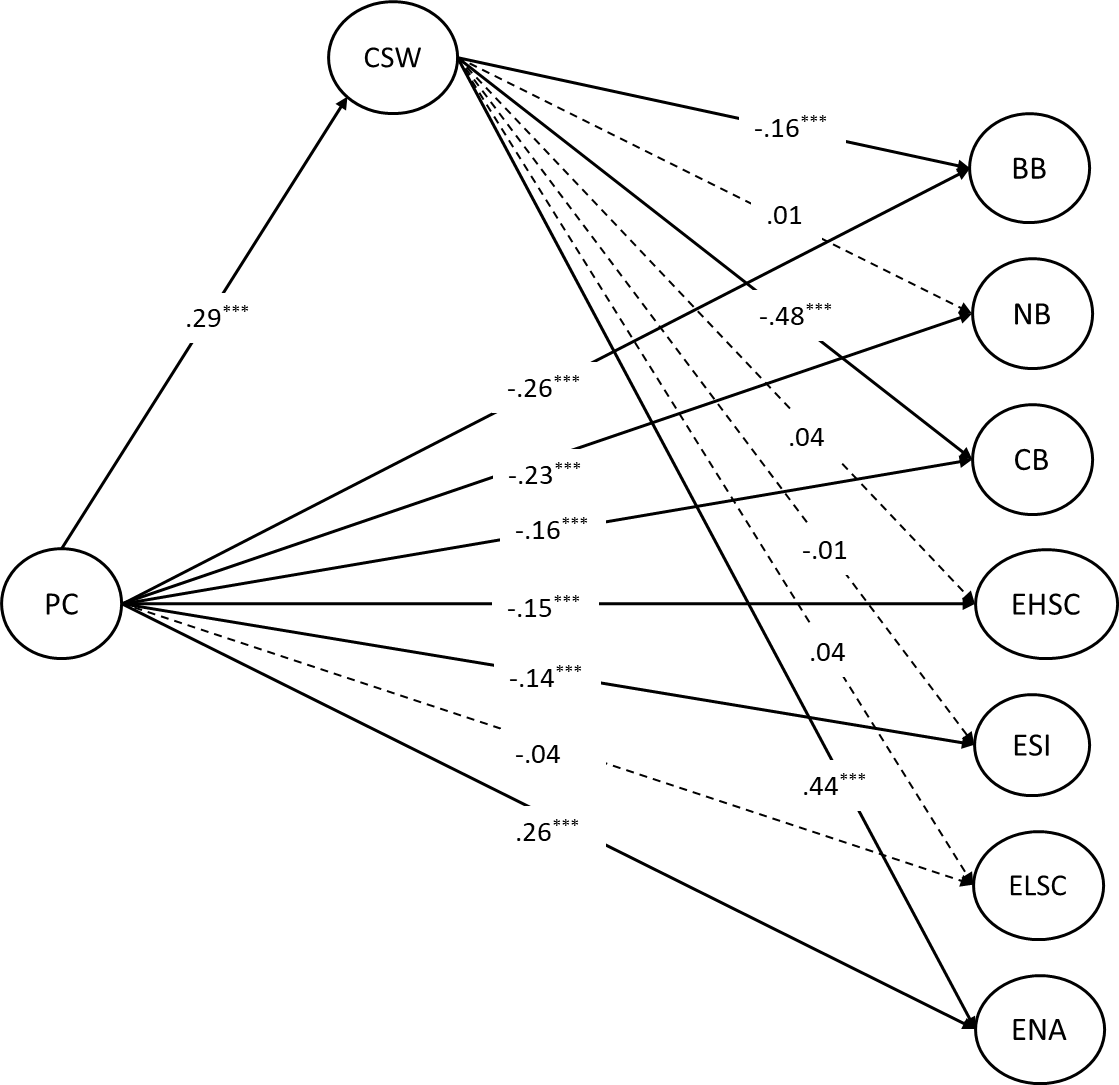


**Figure S5.** Model of perfectionistic concerns predicting beliefs about responding with self-compassion and difficulties enacting self-compassionate responding through contingent self-worth. Path coefficients are standardized. The measurement part of the model, paths representing associations with social desirability, and error terms were estimated but omitted from the figure for clarity. Dashed paths indicate non-significant associations.

*Note*. ^***^*p* < .001. PC = perfectionistic concerns, CSW = contingent self-worth, BB = behavioural beliefs, NB = normative beliefs, CB = control beliefs, EHSC = prototype evaluations of responding with high self-compassion, ESI = prototype evaluations of responding with self-indulgence, ELSC = prototype evaluations of responding with low self-compassion, ENA = difficulties enacting self-compassionate responding.

| **Table S1.** | | |
| --- | --- | --- |
| Indirect effects of perfectionistic concerns on beliefs about responding with self-compassion and difficulties enacting self-compassionate responding through contingent self-worth. | | |
| Indirect Effects Tested | *ab* | 98% CI |
| PC ⇒ Contingent Self-Worth ⇒ Behavioural Beliefs | -.031 | -.054 / -.015 |
| PC ⇒ Contingent Self-Worth ⇒ Normative Beliefs | .001 | -.014 / .019 |
| PC ⇒ Contingent Self-Worth ⇒ Control Beliefs | -.133 | -.172 / -.099 |
| PC ⇒ Contingent Self-Worth ⇒ EHSC | .008 | -.006 / .023 |
| PC ⇒ Contingent Self-Worth ⇒ ESI | -.001 | -.017 / .015 |
| PC ⇒ Contingent Self-Worth ⇒ ELSC | .006 | -.006 / .020 |
| PC ⇒ Contingent Self-Worth ⇒ Enactment Difficulties | .112 | .083 / .147 |

*Note*. PC = perfectionistic concerns, EHSC = prototype evaluations of responding with high self-compassion, ESI = prototype evaluations of responding with self-indulgence, ELSC = prototype evaluations of responding with low self-compassion.
